# Physical Inactivity Mediates the Association between the Perceived Exercising Behavior of Social Network Members and Obesity: A Cross-Sectional Study

**DOI:** 10.1371/journal.pone.0046558

**Published:** 2012-10-02

**Authors:** Janette S. Leroux, Spencer Moore, Lucie Richard, Lise Gauvin

**Affiliations:** 1 School of Kinesiology and Health Studies, Queen’s University, Kingston, Ontario, Canada; 2 Department of Community Health and Epidemiology, Queen’s University, Kingston, Ontario, Canada; 3 Centre de recherche du centre hospitalier de l’ Université de Montréal (CHUM), Montréal, Québec, Canada; 4 Institut de recherche en santé publique de l’Université de Montréal (IRSPUM), Montréal, Québec, Canada; 5 Centre de recherché de l’Institut universitaire de gériatrie de Montréal, Montréal, Québec, Canada; 6 Département de Médecine sociale et préventive, Université de Montréal, Montréal, Québec, Canada; 7 Groupe de recherche interdisciplinaire en santé (GRIS), Faculty of Medicine, Université de Montréal, Montréal, Québec, Canada; The University of Hong Kong, Hong Kong

## Abstract

**Objectives:**

Social networks influence the spread of depression, health behaviors, and obesity. The social networks of older urban-dwelling adults were examined to assess whether physical inactivity mediated the association between social networks and obesity.

**Methods:**

Data come from the Montreal Neighborhood Networks and Healthy Aging study (n = 2707). Self-reported height and weight were used to calculate body mass index (BMI) with obesity defined as a BMI≥30. A name generator/interpreter instrument was used to elicit participants’ core ties (i.e., alters), and assess whether alters exercised regularly and resided in participants’ neighborhoods. The International Physical Activity Questionnaire was used to measure physical inactivity. Separate multilevel logistic regression analyses were conducted for younger (18–54 years) and older (55 years plus) age groups to examine the association between the exercising behavior of alters and obesity. Ancillary analyses examined whether the residential location of alters was associated with obesity. Mediation analyses assessed whether physical inactivity mediated the association between alter exercising behavior and obesity. Models adjusted for participant socio-demographic and -economic characteristics.

**Results:**

Among the older age stratum (55 years plus), physically inactive individuals were more likely obese (OR 2.14; 95% CIs: 1.48–3.10); participants who had more exercising alters were less likely obese (OR: 0.85; 95% CIs: 0.72–0.99). Physical inactivity mediated the association between exercising alters and obesity. Ancillary analyses showed that having exercising alters in the neighborhood compared to other locations tended to reduce the odds of obesity.

**Conclusion:**

This work demonstrates the importance of social networks among older adults in facilitating a physically active lifestyle and reducing the odds of obesity. Such findings can inform the design of public health interventions that seek to improve the environmental conditions supporting the physical activity of older adults.

## Introduction

Recent research on social networks and health has highlighted the importance of networks for the spread of a range of health conditions, including depression and obesity [1], [2]. These studies have shown, for example, that obesity clusters within social networks with close friends and family members influencing a person’s chances of becoming obese [1]. Yet, little is known about the potential behavioral mechanisms by which social networks increase a person’s body mass [1], and whether these mechanisms operate similarly for older and younger adults [3].

Research on social networks and health has identified a number of pathways by which networks may ultimately impact a person’s health. These include behavioral, psychological, and physiological pathways [4]. With regard to behavioral pathways, a person’s social network might presumably impact a person’s physical activity and dietary behavior. Physical activity behavior has been strongly linked to pre-morbid conditions including obesity [5]. Studies on physical activity and obesity have tended to focus on individual physical activity or inactivity levels and the same individual’s risk of obesity, although there is a need for examining the complex interplay between psychosocial and environmental influences on obesity [6], [7], [8]. No research, as far as we are aware, has examined whether the perceived exercising behavior of an individual’s core network members is associated with an individual’s risk of being obese, and whether a person’s own physical activity levels mediates this association. Yet, findings on social networks and obesity have suggested that networks may influence obesity through personal behaviors [1], [9], [10] or via internalized norms on desired body size [11].

Studies of social networks have also shown that the impact of social relationships on health and well-being can vary across the life course. A longitudinal study on networks and aging showed declines in physical and cognitive functioning related to alterations in the size and composition of personal networks [12]. Additional studies investigating the importance of social networks among older adults suggest that increased social ties may be associated with decreased risk of functional decline [13]. Moreover, certain relationships may have been found to reduce subsequent mortality [14]. Compared to older adults, younger adults tend to have larger and more diverse networks via a greater variety of activity settings in which they engage, including workplaces, schools and colleges [10], [15].

Besides the general shrinking of social networks with age, research has also shown that older age groups may be more vulnerable than younger adults to the negative effects of neighborhood environments on health and health-related behavior [4]. Physical environmental features of neighborhoods, such as excessive noise, heavy traffic, and disorder have been identified as risk factors for functional decline [16], [17], [18], [19] and physical activity/inactivity among older adults [20], [21]. Neighborhood social environments, such as poverty, disadvantage and age composition, have been found to be associated with cognitive functioning, mental health, and local park use among older adults [22], [23], [24]. Factors potentially increasing the exposure of older adults to place-based factors include reduced physical mobility to access resources important to health, and the increased importance of localized resources [13], [18], [25]. Such findings suggest that neighborhood settings and the social connections that adults maintain within those settings may be more strongly associated with the health and health-related behavior of older than younger adults. Yet, few studies have assessed whether the neighborhood social ties of older compared to those of younger adults are more salient influences on health and health-related behavior.

Using network data on the composition of adult social networks, the following study addresses three sets of questions:

1. Does the potential association between perceived exercising behavior of a person’s core network and their obesity status differ by older and younger age stratum?
2. Does an individual’s own physical activity level mediate the association between obesity and the perceived exercising behavior of a person’s core network?
3. Is the perceived exercising behavior of core members residing in the neighborhood more strongly associated with obesity compared to those residing elsewhere?

## Methods

### Ethics Statement

Prior to taking part in the study’s telephone interview, participants were read the study’s letter of information and consent form. If individuals gave verbal consent to participate, they were administered the questionnaire by trained interviewers. Verbal consent was recorded on the computer-administered telephone interviewing system. This interview protocol was documented in the study’s ethics application. Ethics approval for the study was given by the Committee of Scientific Evaluation and Research Ethics of the Centre de Recherche at the Centre Hospitalier de l’Université de Montréal (CHUM) in October 2007 (N.D. 07.049).

### Sample

Data come from the Montreal Neighborhood Networks and Health Aging Study (MoNNET-HA). The MoNNETs-HA study used a two-stage stratified cluster sampling design. In stage one, Montreal Metropolitan Area (MMA) census tracts (N = 862) were stratified using 2001 Canada Census data into tertiles of high, medium, and low household income. One hundred census tracts were selected from each tertile (n_j_ = 300). In stage two, potential household respondents within each tract were stratified into three age groups: 25–44 years old, 45–64, and 65 or older. Three respondents were randomly selected within each age stratum and census tract for a total of 9 respondents per tract, except for seven tracts in which four participants were selected (n_i_ = 2707). To be selected, individuals had to 1) be non-institutionalized, 2) have resided at their current address for at least one year, and 3) able to complete the questionnaire in French or English. Random digit dialing of listed telephone numbers was used to select households and a computer-assisted telephone interviewing system guided questionnaire administration. Participants completed the telephone interview between mid June and early August 2008.

MoNNET-HA had a response rate of 38.7%. Chi-square analyses showed the MoNNET-HA sample to over represent 1) older adults (by sampling design), 2) individuals with an income less than 50,000 per year, 3) persons who lived in their places of residence for more than five years, 4) females, and 5) those with more than a high school degree.

### Measures

#### Obesity

Participants reported their height in meters and weight in kilograms. This information was used to estimate body mass index (BMI) (kg/m2) of each participant. Obesity status was defined as a BMI>30 kg/m2.

#### Physical inactivity

Participants reported their physical activity levels using the short-form version of the International Physical Activity Questionnaire (IPAQ). The IPAQ incorporates questions about the total volume of physical activity and the number of days during a week that such activity was conducted to calculate the energy costs of activity as the metabolic equivalent of task (MET). For example, participants would be asked “*In a typical week in the past four weeks, did you walk fast or briskly? If yes, how many total hours a week did you normally do this?*”, with responses categorized in 1.5 hour increments. IPAQ guidelines were used to classify respondents according to activity type and MET into high/moderate or low/inactive physical activity levels. Individuals were classified as being physically inactive if they did not attain the levels of moderate or high physical activity [26]. For this analysis, physical inactivity was a dichotomous variable representing the low or inactive level compared to moderate and high levels.

#### Social network measures

To identify respondents’ core social networks, MoNNET-HA asked participants to name up to three people (i.e., their alters) with whom they had discussed important matters in the past six months. A name interpreter instrument followed the name generator question. The name interpreter consisted of a series of questions asking about those alters. One question asked participants whether their alters did or did not exercise regularly. The “exercising alters” variable represented the number of alters that a participant reported as exercising regularly. A second question asked participants if their alters resided in their i) household, ii) neighborhood, iii) in the MMA, or iv) outside the MMA.

#### Confounding variables

To adjust for the potential confounding effects of age on network characteristics, physical inactivity, and obesity, analyses were stratified into younger (25–54 years) and older (55+ years) age groups. Within each age stratum, analyses also adjusted for the following age categories: 25–34, 35–44, 45–54, 55–64, 65–74, and 75 or older. Analyses also adjusted for gender, socioeconomic status, marital status, and household language. Socioeconomic status was based on a composite factor score consisting of educational attainment, household income, and employment status. Marital status was classified as married/common-law relationship, single, divorced, and widowed. Household language was grouped into French, English, or other.

### Statistical Analyses

#### Statistical analysis procedures

Separate multilevel logistic regression analyses were conducted for younger and older age strata. Multilevel methods were used to account for MoNNET’s clustered sampling design, and assess the associations among obesity, physical inactivity, network characteristics, and potential confounders. The following series of analyses were conducted. First, in model one, obesity was regressed on participant socioeconomic and demographic characteristics and the number of alters who exercise. Second, obesity was regressed on participant socioeconomic and demographic characteristics and the number of alters who exercised in the household, neighborhood, MMA, and outside the MMA. Third, obesity was regressed on previous variables and participant physical inactivity levels. Formal mediation analyses were then conducted to assess whether a participant’s physical inactivity behavior mediated the association of obesity and exercising alters. Observations were excluded if they were missing information on any study variables.

Multilevel regression and the three-step mediation analysis procedures recommended by Krull and MacKinnon were followed [27]: (1) physical inactivity was regressed on exercising alters with the estimated association between the two represented using ***a***; (2) obesity was regressed on exercising alters and physical inactivity with the estimated association between obesity and physical inactivity represented using ***b***; (3) the two coefficients were then multiplied together (***ab***) to provide an estimate of the mediated effect of exercising alters. First-order Taylor series expansion was used to provide estimates of the standard error of the mediated effect [26]. The ratio of the ***ab*** estimate and its standard error were used to calculate z-scores, Wald statistics, and 95% confidence intervals to test the null hypothesis that the ***ab*** estimate of the mediation effect was zero. All estimates were adjusted for the participants’ socio-demographic and -economic characteristics. Due to convergence problems, we were unable to assess the role of exercising alter’s residential proximity in the mediation analyses.

## Results

Table 1 provides descriptive information on the study sample. Among the younger adult sample, obesity and physical inactivity had prevalence rates of 14.5% and 12.6% respectively. Approximately 20.7% of younger adults reported having no alter who exercised regularly. Among older adults, obesity and physical inactivity had prevalence rates of 16.0% and 21.9%. Roughly thirty-three percent of older adults reported not having an alter who exercised regularly. Tables 2 and 3 provide the results of the stratified multilevel logistic analyses; Table 4 provides the results of the mediation tests.

**Table 1:** MoNNET-HA Participant Characteristics, n = 2558.

Variable	Younger Stratum, n = 1329	Older Stratum, n = 1229
Obese	14.5%	16.0%
Physically inactive	12.6%	21.9%
Age Group		
25–34	28.3%	–
35–44	33.4%	–
45–54	38.3%	–
55–64	–	33.7%
65–74	–	44.3%
75+	–	22.0%
No exercising alters	20.7%	33.5%
Exercising alters (at least one)		
in Household	20.5%	11.1%
in Neighborhood	30.2%	27.2%
in MMA	26.3%	20.7%
outside MMA	39.3%	36.2%
Female	62.9%	65.3%
Educational attainment		
No degree	4.7%	19.9%
High school/Trade	24.7%	33.5%
College	25.8%	15.1%
University	44.8%	31.4%
Household Income		
<28,000	11.9%	29.3%
28,000–49,000	26.8%	29.9%
50,000–74,000	28.5%	25.3%
75,000–100,000	16.0%	9.4%
>100,000	16.9%	6.1%
Employed	83.2%	23.7%
Marital status		
Married/Common Law	61.0%	47.0%
Single	26.7%	13.4%
Separated	4.5%	3.8%
Divorced	6.7%	15.2%
Widowed	1.1%	20.5%
Household Language		
French	75.9%	81.1%
English	13.5%	13.4%
Other (Bilingual/Other)	10.7%	5.5%

**Table 2:** Younger age stratum, adjusted odds ratios and 95% confidence intervals.

Variable	Younger Age Stratum, n = 1329		
	Model 1	Model 2	Model 3
**Age Category**			
25–34 yrs (ref.)	1.00	1.00	1.00
35–44 yrs	1.78 (1.14–2.78)	1.76 (1.13–2.74)	1.73 (1.11–2.70)
45–54 yrs.	2.24 (1.45–3.48)	2.18 (1.41–3.36)	2.13 (1.38–3.29)
**No. Exercising alters**	–	1.03 (0.88–1.20)	1.04 (0.89–1.22)
in household	0.92 (0.62–1.36)	–	–
in neighborhood	1.16 (0.93–1.45)	–	–
in MMA	0.79 (0.58–1.06)	–	–
outside MMA	1.08 (0.87–1.34)	–	–
**Physical inactivity**	–	–	1.54 (1.01–2.35)
**Gender**			
Female	0.63 (0.46–0.87)	0.64 (0.47–0.88)	0.64 (0.46–0.88)
Male (ref.)	1.00	1.00	1.00
**Socioeconomic** **Status**	0.58 (0.45–0.73)	0.57 (0.45–0.72)	0.58 (0.46–0.74)
**Marital status**			
Single	0.89 (0.61–1.32)	0.90 (0.62–1.31)	0.90 (0.62–1.32)
Separated	0.74 (0.34–1.64)	0.75 (0.34–1.65)	0.78 (0.35–1.72)
Divorced	0.64 (0.33–1.25)	0.63 (0.32–1.23)	0.63 (0.32–1.23)
Widowed	1.25 (0.37–4.17)	1.43 (0.44–4.72)	1.41 (0.43–4.63)
Married/Common Law(ref.)	1.00	1.00	1.00
**Household Language**			
English	1.35 (0.87–2.10)	1.33 (0.86–2.07)	1.33 (0.85–2.07)
Foreign Language	0.93 (0.54–1.61)	0.91 (0.53–1.56)	0.88 (0.51–1.51)
French (ref.)	1.00	1.00	1.00

**Table 3:** Older age stratum, adjusted odds ratios and 95% confidence intervals.

Variable	Older Age Stratum, n = 1229		
	Model 1	Model 2	Model 3
**Age Category**			
55–64 yrs.	1.32 (0.80–2.18)	1.31 (0.80–2.16)	1.41 (0.84–2.34)
65–74 yrs.	1.51 (0.98–2.35)	1.50 (0.97–2.31)	1.54 (0.98–2.40)
75 & older (ref.)	1.00	1.00	1.00
**No. Exercising alters**	–	0.82 (0.70–0.95)	0.85 (0.72–0.99)
in household	0.72 (0.42–1.22)	–	–
in neighborhood	0.74 (0.57–0.97)	–	–
in MMA	0.78 (0.57–1.07)	–	–
outside MMA	0.87 (0.70–1.07)	–	–
**Physical inactivity**	–	–	2.14 (1.48–3.10)
**Gender**			
Female	0.91 (0.65–1.28)	0.90 (0.64–1.26)	0.82 (0.58–1.16)
Male (ref.)	1.00	1.00	1.00
**Socioeconomic** **Status**	0.83 (0.65–1.07)	0.82 (0.64–1.06)	0.88 (0.68–1.13)
**Marital status**			
Single	1.15 (0.71–1.87)	1.17 (0.72–1.88)	1.13 (0.69–1.84)
Separated	1.01 (0.44–2.29)	1.03 (0.45–2.34)	1.08 (0.47–2.49)
Divorced	0.89 (0.55–1.46)	0.91 (0.56–1.47)	0.97 (0.59–1.60)
Widowed	0.97 (0.61–1.55)	0.99 (0.62–1.57)	0.98 (0.61–1.57)
Married/Common Law(ref.)	1.00	1.00	1.00
**Household Language**			
English	1.15 (0.72–1.83)	1.13 (0.71–1.80)	1.07 (0.67–1.72)
Foreign Language	1.05 (0.54–2.06)	1.03 (0.53–2.02)	0.94 (0.47–1.88)
French (ref.)	1.00	1.00	1.00

**Table 4:** Mediation Tests.

Mediation test	a(standard error)	b(standard error)	ab(standard error)	95% Confidence Intervals	Waldstatistic
1. Younger age stratum (25–54 yrs. old), n = 1329	−0.33***(0.09)	0.43*(0.22)	−0.14(0.08)	−0.30, 0.02	3.12
2. Older age stratum (55 yrs. and older), n = 1229	−0.30*** (0.07)	0.76*(0.19)	−0.23**(0.08)	−0.38, −0.08	8.66

* p<0.05,

** p<0.01,

*** p<0.001.

(1) ***a:*** coefficient representing the estimated association of physical inactivity (*y*) with perceived exercising alters (*x*).

(2) ***b***: coefficient representing the estimated association of obesity (*y*) with physical inactivity (*x*).

(3) ***ab***: product of the two coefficients, representing an estimate of the mediated effect.

### Younger Age Stratum

Among adults between 25–54 years old, adults within the age categories of 35–44 years (OR: 1.73; 95% CIs: 1.11–2.70) and 45–54 years (OR: 2.13; 95% CIs: 1.38–3.29) were more likely to be obese than 25–34 years old. Females (OR: 0.64; 95% CIs: 0.46–0.88) were less likely to be obese than males. Higher SES persons (OR: 0.58; 95% CIs: 0.46–0.74) were less likely to be obese. Neither the number nor the residential location of perceived exercising alters was associated with obesity. Physical inactivity (OR: 1.54; 95% CIs: 1.01–2.35) increased the odds of obesity. Having a higher number of perceived exercising alters reduced the likelihood of physical inactivity among younger adults (***a*** = −0.33, *p<*0.001). Physical inactivity did not however mediate the association between obesity and the exercising behavior of alters.

### Older Age Stratum

Among adults older than 55 years, there were no differences among age categories in the risk of being obese. Having a higher number of exercising alters reduced an older adult’s chances of being obese (OR: 0.85; 95% CIs: 0.72–0.99). Physical inactivity increased the likelihood of obesity (OR: 2.14; 95% CIs: 1.48–3.10). Mediation analyses showed that physical inactivity partially mediated the association between exercising alters and obesity (**ab** = −0.23; SE = 0.08; p<0.01). Ancillary analyses showed that having exercising alters in the neighborhood reduced the odds of obesity among older adults (OR: 0.74; 95% CIs: 0.57–0.97). No other residential location was associated with obesity.

## Discussion

Our study addressed three questions regarding the association of obesity, physical inactivity, and social networks in urban-dwelling adults. First, the study found that the association between a person’s obesity status and the perceived exercising behavior of their core network did differ by older and younger age stratum. Although no association was found in younger adults, there was an association shown between obesity and the social network characteristics of older adults. In contrast, SES was a significant factor for obesity among younger and not older adults. Such findings may highlight the relatively increased importance of network resources in the health status of older age groups. Second, in each age stratum, there was an association observed between the perceived number of exercising alters and the participant’s physical inactivity level. Yet, only among older adults did physical inactivity partially mediate the association between obesity and the exercising behavior of alters. As such, it is important to note, firstly, that physical inactivity explained most of the association between social networks and obesity among older adults. Nevertheless, there was some remaining association between networks and obesity, suggesting that having alters who exercise may confer obesity-prevention advantages beyond those of physical activity (e.g., healthy dietary advice). The lack of mediation in younger adults may be reflective of broader network influences (i.e., beyond core ties) at play in younger age groups, their greater exposure to a range of social contexts, or a stronger role for other environmental or behavioral correlates of obesity. Third, the ancillary analyses showed that among older adults, the strongest association between obesity and exercising alters was found for those whose exercising alters resided in the participants’ neighborhoods. The lack of an association in the other residential categories (e.g., outside Montreal) suggests that neighborhood-dwelling alters may have a relatively greater role to play in the physical activity behavior and obesity status of older adults. Recent research on neighborhoods, health, and older adults has emphasized the potential importance of residential settings for the physical, social and emotional health of older adults [15], [28], [29], [30]; physical and cognitive decline causes older adults to be more vulnerable and potentially more reliant on spatially proximate built and social environmental resources [4]. Our study supports this research, suggesting that neighborhood social resources may play an important role in the physical activity behavior and obesity of older adults.

This study does have limitations. First, the study’s cross-sectional nature prohibits causal inferences from being drawn. In terms of network analysis, this precludes the opportunity to disentangle social selection from influence. In other words, rather than assuming that the exercising alters somehow altered participant physical activity levels, it may be that participants sought out alters who tended to be like themselves in terms of physical activity patterns, a process often referred to as homophily [10]. Second, our study focused on the importance of core network ties to health behaviors and outcomes, and did not extend beyond core ties, including weak, acquaintance ties and their possible association with physical inactivity and obesity in adults. It may be possible that weak compared to strong ties play a greater role in the physical activity patterns of younger adults [9], [30]. Finally, given the population-based aspect of the study, costs and feasibility dictated the use of self-reported outcome measures of BMI and physical activity, as well as non-objective measures of peer physical activity. Physically inactive participants may in fact over-report their alters as not exercising from a ‘false consensus effect’, whereby individuals are more likely to perceive themselves as similar to others for attributes which are considered socially undesirable [31]. Further studies that assess covariance in the potential underreporting of physical inactivity and weight status are needed to determine whether reporting biases would alter the associations reported.

Despite these limitations, this study expands on research examining the spread of health behaviors and outcomes through social networks in several meaningful ways. Our analysis offers a unique perspective on the potential pathways linking networks to obesity among older adults. More specifically, we highlight the potential importance of behavioral pathways, particularly physical inactivity, in the link between networks and obesity in older adults. An increasingly sedentary lifestyle and expanding aging population carries widespread implications for an already over-burdened healthcare system, and creates an urgency to investigate the complex interplay between psychosocial and environmental factors which contribute to physical activity behavior [7], [8]. Our study shows that neighborhood social resources play a relatively important role for older adults. The current findings have potentially significant implications for health policy and community-based interventions aimed at addressing the alarming rate of physical inactivity among older adults, and averting various adverse health outcomes related to physical inactivity and obesity (osteoporosis, chronic disease risk factors and events, physical and functional disability, and institutional aging) within this demographic [5]. As such, our study suggests that neighborhood settings may be critical sites for the design and implementation of health promotion programs that target ecological factors, e.g., local social networks, in the effort to improve older adult physical activity levels and health outcomes.
